# Battling Chemotherapy-induced Nausea and Vomiting in Cancer Palliative Care

**DOI:** 10.4103/0973-1075.78457

**Published:** 2011

**Authors:** Dilip Gude

**Affiliations:** AMC, 3^rd^ Floor, Medwin Hospital, Chirag Ali lane, Nampally, Hyderabad 500 001, AP, India

Sir,

Chemotherapy-induced nausea and vomiting (CINV) is a considerably distressing symptom commonly faced by a large group of patients receiving cancer therapy. CINV can be classified as either acute occurring within the first 24 h of chemotherapy administration or delayed occurring between 24 h and 5 days post-chemotherapy. Inadequately controlled CINV is associated with higher medical costs, significant loss of the patient’s ability to work, and decreased quality of life.

Multinational Association of Supportive Care in Cancer (MASCC) has classified the emetogenic potential of chemotherapy agents as high, moderate, low, and minimal. Doxorubicin or epirubicin with cyclophosphamide, carmustine >250 mg/m^2^, cisplatin ≥50 mg/m^2^, cyclophosphamide >1500 mg/m^2^, dacarbazine, mechlorethamine, and streptozocin are grouped among the highly emetic chemotherapy agents. The current recommendations when dealing with such highly emetogenic drugs are to give triple therapy with a 5-HT3 receptor antagonist, dexamethasone, and neurokinin-1 receptor (NK1) antagonist on day 1 before chemotherapy is begun followed by dexamethasone and aprepitant on days 2 and 3, and dexamethasone alone on day 4[1] Breakthrough emesis (vomiting that occurs on any day of treatment despite the administration of optimal antiemetic prophylaxis) is something that the clinician needs to be geared up for and it is generally suggested to administer anitemetics from classes other than those the patient is currently on. Drugs such as dopamine antagonists, haloperidol, and benzodiazepines can be added. Cannabinoids such as nabilone and dronabinol can be reserved for persisting CINV even after the conventional antiemetic treatment. Concomitant acid suppression can also help alleviate the symptoms.

Palonosetron has a higher binding affinity for the 5-HT3 receptor than do the first-generation agents (ondansetron and granisetron), and it has a longer half-life, exerting its effect for several days after a single administration.[2] In various studies, the NK1 receptor antagonists (belonging to the class-substance P antagonists) casopitant, aprepitant, and fosaprepitant (a water-soluble phosphoryl prodrug of aprepitant) have shown their efficacy in ameliorating CINV.[3] Aprepitant improves CINV regardless of various risk factors such as younger age, female gender, CINV in prior cycles, etc. In the efficacy of controlling CINV, the atypical antipsychotic olanzapine was comparable to aprepitant. Patient expectancy of nausea and vomiting (about 20% by the fourth cycle) is an under-looked factor and manipulations such as behavioral therapies (progressive muscle relaxation training, systematic desensitization, hypnosis, and relaxation with music and imagery) can be used to effectively treat anticipatory CINV.[4] Combining pressure point-P6 acupressure with antiemetics has shown better results than either one alone. Ginger supplementation[5] and intake of flavonoid-rich fruits and vegetables have also shown to improve CINV.

Patient factors such as polypharmacy, medication costs, mucositis, and depression and factors on the healthcare professionals’ part such as heavy workloads (lack of time and empathy), poor communication, and underestimation of the problem hinder achieving a reasonable antiemetic control. Assessment and modification of risk factors, adherence to guidelines and antiemetic regimens, and the use of novel strategies such as long-acting formulations, ambulatory pumps, transdermal patches, and nasal sprays may improve patient outcomes and enhance the quality of life.
